# Aminophylline Induces Two Types of Arrhythmic Events in Human Pluripotent Stem Cell–Derived Cardiomyocytes

**DOI:** 10.3389/fphar.2021.789730

**Published:** 2022-01-17

**Authors:** Simon Klimovic, Martin Scurek, Martin Pesl, Deborah Beckerova, Sarka Jelinkova, Tomas Urban, Daniil Kabanov, Zdenek Starek, Marketa Bebarova, Jan Pribyl, Vladimir Rotrekl, Kristian Brat

**Affiliations:** ^1^ CEITEC, Masaryk University, Brno, Czechia; ^2^ Department of Biology, Faculty of Medicine, Masaryk University, Brno, Czechia; ^3^ Department of Biochemistry, Faculty of Science, Masaryk University, Brno, Czechia; ^4^ Department of Respiratory Diseases, University Hospital Brno, Brno, Czechia; ^5^ Faculty of Medicine, Masaryk University, Brno, Czechia; ^6^ International Clinical Research Center, St. Anne’s University Hospital, Brno, Czechia; ^7^ First Department of Internal Medicine—Cardioangiology, Faculty of Medicine, St. Anne’s University Hospital, Masaryk University, Brno, Czechia; ^8^ Department of Physiology, Faculty of Medicine, Masaryk University, Brno, Czechia

**Keywords:** aminophylline, IPSC, hESC, cardiomyocytes, drug cardiotoxicity, atomic force microscopy, arrhythmogenic effects, methylxanthines

## Abstract

Cardiac side effects of some pulmonary drugs are observed in clinical practice. Aminophylline, a methylxanthine bronchodilator with documented proarrhythmic action, may serve as an example. Data on the action of aminophylline on cardiac cell electrophysiology and contractility are not available. Hence, this study was focused on the analysis of changes in the beat rate and contraction force of human pluripotent stem cell–derived cardiomyocytes (hPSC-CMs) and HL-1 cardiomyocytes in the presence of increasing concentrations of aminophylline (10 µM–10 mM in hPSC-CM and 8–512 µM in HL-1 cardiomyocytes). Basic biomedical parameters, namely, the beat rate (BR) and contraction force, were assessed in hPSC-CMs using an atomic force microscope (AFM). The beat rate changes under aminophylline were also examined on the HL-1 cardiac muscle cell line via a multielectrode array (MEA). Additionally, calcium imaging was used to evaluate the effect of aminophylline on intracellular Ca^2+^ dynamics in HL-1 cardiomyocytes. The BR was significantly increased after the application of aminophylline both in hPSC-CMs (with 10 mM aminophylline) and in HL-1 cardiomyocytes (with 256 and 512 µM aminophylline) in comparison with controls. A significant increase in the contraction force was also observed in hPSC-CMs with 10 µM aminophylline (a similar trend was visible at higher concentrations as well). We demonstrated that all aminophylline concentrations significantly increased the frequency of rhythm irregularities (extreme interbeat intervals) both in hPSC-CMs and HL-1 cells. The occurrence of the calcium sparks in HL-1 cardiomyocytes was significantly increased with the presence of 512 µM aminophylline. We conclude that the observed aberrant cardiomyocyte response to aminophylline suggests an arrhythmogenic potential of the drug. The acquired data represent a missing link between the arrhythmic events related to the aminophylline/theophylline treatment in clinical practice and describe cellular mechanisms of methylxanthine arrhythmogenesis. An AFM combined with hPSC-CMs may serve as a robust platform for direct drug effect screening.

## Introduction

Theophylline (1,3-dimethylxanthine) and its more soluble form aminophylline (a complex of two theophylline molecules and ethylenediamine) are well-known bronchodilators used mostly for therapy of chronic obstructive pulmonary disease (COPD) and asthma. These drugs are also recommended for the treatment of emphysema (Zatloukal et al., 2020). Other indications have been suggested, for example, treatment of apnea in premature neonates (Ye et al., 2019). Unfortunately, a narrow therapeutic range and frequent adverse effects make their use controversial (Singh et al., 2019). An increased mortality rate in theophylline users has been reported by multiple research teams (Lee et al., 2009; Horita et al., 2016). A higher percentage of cardiovascular deaths was observed in asthma patients who received aminophylline (Suissa et al., 1996), as well as in patients with COPD (Lee et al., 2009), and also in a general patient population overdosed with theophylline (Shannon, 1999).

The adverse effects of theophylline/aminophylline include arrhythmias, even at their therapeutic plasma concentrations (Bittar and Friedman, 1991). In a meta-analysis of several randomized clinical trials, 13% incidence of arrhythmias or palpitations after intravenous aminophylline infusion has been observed (Nair et al., 2012). More than 20% of patients experience cardiac arrhythmias during an aminophylline overdose episode (Shannon, 1999). Supraventricular arrhythmias have been observed most frequently, usually represented by atrial fibrillation (AF) (Varriale and Ramaprasad, 1993; Huerta et al., 2005). Concurrently, COPD is an independent risk factor of AF (Goudis, 2017). The risk of arrhythmias is further enhanced by hypokalemia (Hoppe et al., 2018), which may develop as a side effect of aminophylline treatment, particularly occurring in cases of intentional overdose (Ellis, 1985; Charytan and Jansen, 2003).

Mechanisms of theophylline-/aminophylline-induced arrhythmias are not clear. It is well known that methylxanthines non-specifically inhibit phosphodiesterases (hence increasing the cAMP level) and adenosine receptors (Ukena et al., 1993). These effects may explain the sinus tachycardia often observed in clinical practice. In contrast, this does not explain the origin of AF associated with aminophylline treatment. Effects of theophylline/aminophylline on cardiac electrophysiology were studied mostly in animal models (Komadina et al., 1992; Onodera et al., 2001; Shamsuzzaman et al., 2016). However, a conclusive explanation of their proarrhythmic action has not been provided, and data from human cardiomyocytes (CMs) embedded in cardiac syncytia have been missing completely.

**FIGURE 1:**
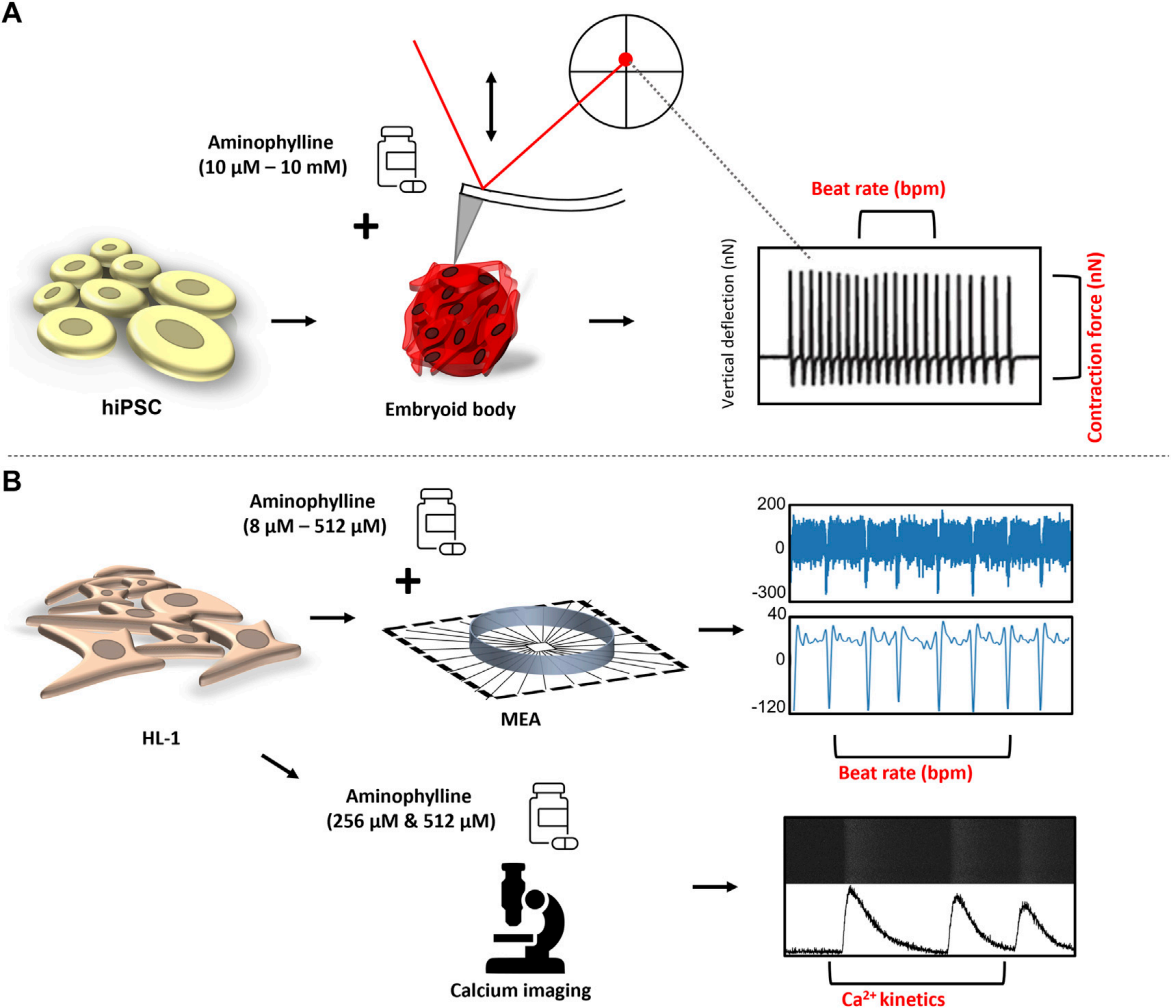
Experimental design: **(A)** hPSCs were differentiated in the form of EBs into contracting 3D clusters of cardiomyocytes, and under the influence of increasing concentrations of aminophylline, the mechanocardiogram (MCG) was recorded was recorded by an atomic force microscope. The BR and contraction force values of each contractive event were further analyzed and compared. **(B)** Field potential by the multielectrode array (MEA) system, together with intrinsic Ca^2+^ kinetics via Fluo-8 AM, a calcium-binding dye, was assessed on HL-1 cells to further confirm the arrhythmogenic effect of aminophylline.

This study was aimed to reveal changes of basic functional characteristics (contraction force and beat rate) of human cardiac tissues induced by aminophylline at a wide range of concentrations. A clinically relevant model was employed, for example, 3D structures called embryoid bodies (EBs) derived from human pluripotent stem cells subsequently differentiated into cardiomyocytes (hPSC-CMs). The model was originally described in previous studies (Burridge et al., 2012; Pesl et al., 2014). Its functional properties were further validated using atomic force microscopy (Liu et al., 2012; Dinarelli et al., 2018; Pribyl et al., 2019), a method enabling real-time monitoring of contractions and thus also beating properties of single cardiomyocytes or cardiac cell clusters, alone or in combination with other methods such as microelectrode array (Caluori et al., 2019b) or calcium imaging (Odstrcilik et al., 2015; Caluori et al., 2019a). This model was recently used for disease modeling in studies by Acimovic et al., 2018; Jelinkova et al., 2019. The effects of aminophylline were further validated on an independent model of HL-1 cardiomyocyte cells. The overall scheme of all experiments within this study is presented in Figure 1. This approach has provided the first experimental data well-corresponding with the aminophylline-induced arrhythmias observed in clinical medicine.

## Materials and Methods

### Cell Cultivation

The hESC line CCTL14 (46 XX) derived at Masaryk University, Brno, and previously characterized (International Stem Cell Initiative et al., 2007) was routinely maintained on a feeder layer of mitotically inactivated mouse embryonic fibroblasts as previously described (Dvorak et al., 2005; Krutá et al., 2014; Jelinkova et al., 2020).

Differentiation into CMs via embryoid bodies (EBs) and measurements by an AFM were performed as previously described (Pesl et al., 2014), with minor modifications. Briefly, hESC colonies were collected 4 days after seeding and subsequently broken down into smaller clumps which were seeded into the EB medium (86% KO DMEM, 10% FBS, 1% L-glutamine, 1% penicillin/streptomycin, 1% non-essential amino acids, 0.1 mM 2-mercaptoethanol, and 10 µg/ml ascorbic acid) with 10 ng/ml BMP4 (R&D) and placed in hypoxic conditions (5% O_2_, 5% CO_2,_) where they spontaneously formed EBs. After 3 days, the medium was removed and replaced with fresh EB medium supplemented with 5 ng/ml FGF2 (Peprotech), 10 ng/ml BMP4, and 6 ng/ml activin A (R&D) for a 4-day incubation. A 3-day incubation in EB medium supplemented with 10 ng/ml VEGF (R&D) and 10 µM IWR1 (R&D). The next induction medium was the EB medium supplemented with 10 ng/ml VEGF and 5 ng/ml FGF2, with a medium exchange every 4 days. After four days in this medium (day 14 of differentiation), the EBs were transferred into a normoxic incubator (21% O_2_, 5% CO_2_) for the remaining 8 days of this induction period. From then onward, EBs remained in normoxia and were fed with the EB medium every 4 days until analysis. Beating EBs were selected and transferred on a gelatin-coated PM3 dish to adhere to for measurement. These cellular constructs of the cardiac syncytium were coupled to an AFM force sensor to perform a high-fidelity contraction pattern as an hPSC-CM–based biosensor (Pesl et al., 2014). The constant size of clusters of hPSC-CMs allows comparable and stable beating pattern evaluation allowing for force- and rhythm-related drug effect tracking.

For the immunocytochemistry experiment, either whole or dissociated EBs attached on coverslips were fixed with 4% PFA, blocked and permeabilized by 1% BSA (Sigma) in 0,1% Triton-X (Sigma) or 0,05% TWEEN (Sigma) in PBS, and incubated with anti–troponin T antibody (1:5,000, Cell Signaling, 5,593, Rb) overnight in 4 C. Anti-rabbit Alexa 594 (1:500, Invitrogen) was left to incubate for an hour at room temperature, and the slips were mounted on slides with Mowiol containing DAPI (Sigma). The images were obtained using a Zeiss LSM 700 confocal microscope.

The contracting clusters were previously checked for expression and checked by immunostaining for cardiac troponin T (cTnT) and ryanodine receptor RyR2 as described elsewhere (Pesl et al., 2014; Souidi et al., 2021). The expression of myosin heavy chain MYH6/7 and MYH7, RyR2, and the striated pattern of cTnT in dissociated cardiomyocytes was used to assess the progress in CM maturation. The clusters consisted of all three CM subtypes as described elsewhere; in brief, nodal-like CMs had an AP duration at 90% of repolarization shorter than 100 ms (about 16%), slightly more frequent were atrial-like cells, and recorded AP mainly demonstrated a typical ventricular-like shape (Acimovic et al., 2018). EB responses to basic heart modulators, such as β adrenoceptor agonist and blocker, isoproterenol, and metoprolol, were previously measured (Supplementary Figure S1).

**FIGURE 2:**
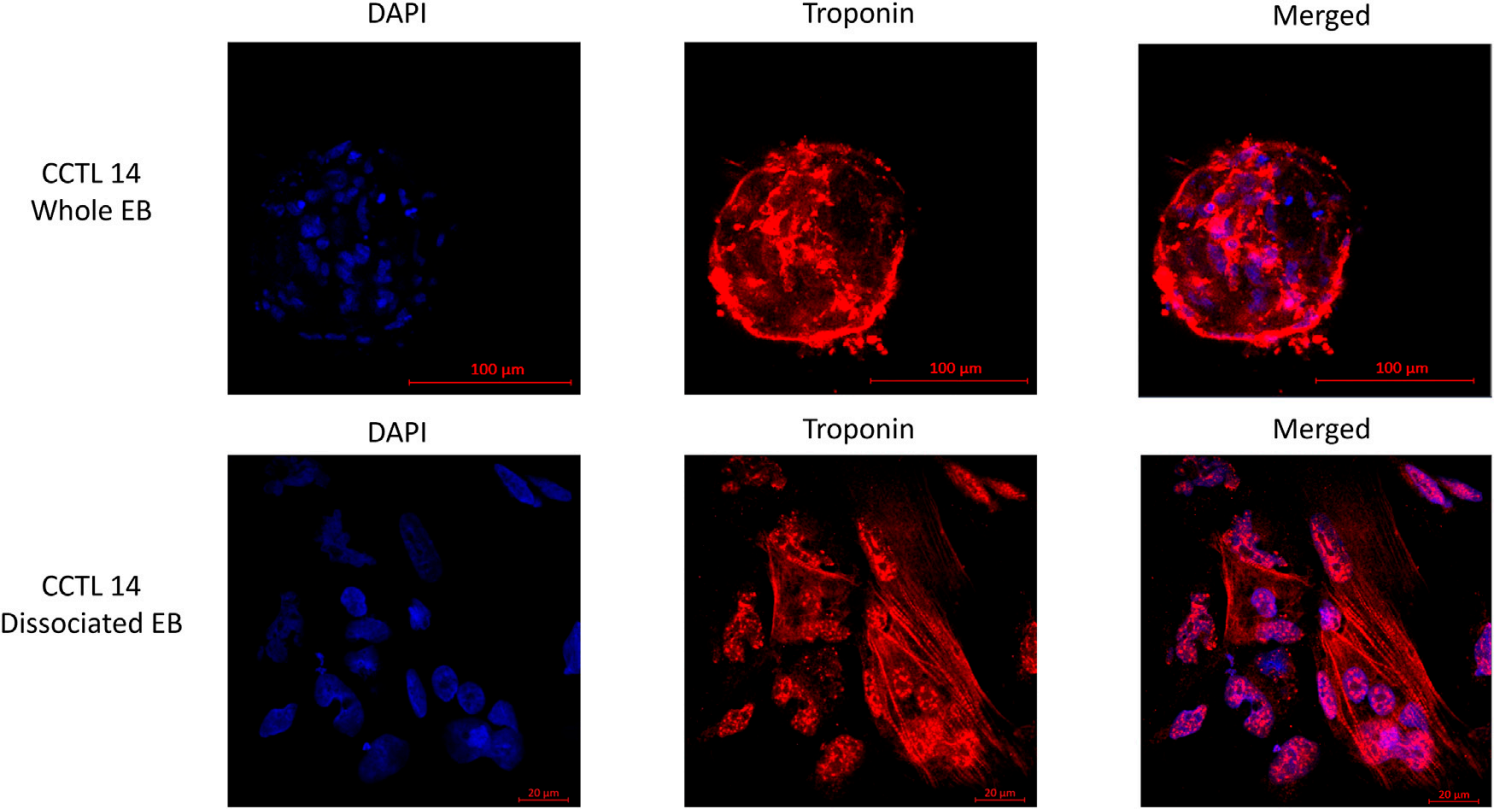
Fluorescence staining of whole and dissociated CCTL14 EBs (cell cluster) illustrates the presence of matured cardiomyocytes identified by the presence of troponin T (red color); cells are localized with DAPI staining of the nucleus (blue color).

EBs from the same cultivation batch were checked for the presence of cTnT in the clusters as well as in the dissociated EBs by immunostaining to demonstrate the batch-to-batch differentiation consistency (Figure 2).

The HL-1 Cardiac Muscle Cell Line (Sigma) was routinely maintained on fibronectin-coated dishes in Claycomb medium (Sigma) supplemented with 10% FBS, 100 U/ml:100 µg/ml penicillin:streptomycin, 0.1 mM norepinephrine, and 2 mM L-glutamine. The cells were passaged with trypsin after reaching full confluency, usually every 3–4 days, in a ratio of 1:3. For experiments with MEA, the HL-1 cells were cultivated on MEA chips coated with fibronectin in 0.02% gelatin (5 mg/ml; Sigma). The HL-1 response to β-adrenoceptor agonist isoproterenol was measured (Supplementary Figure S1).

### Atomic Force Microscopy Measurements

NanoWizard 3 AFM (Bruker-JPK) combined with inverted light microscope IX-81 (Olympus) was used to obtain mechanocardiograms (MCGs) of beating EBs as previously described (Pesl et al., 2014; Caluori et al., 2019b; Pribyl et al., 2019).

Drug response tests were performed after initial equilibration of EBs in Tyrode’s solution (composition in mM: NaCl 135, KCl 5.4, MgCl_2_ 0.9, CaCl_2_ 1.8, HEPES 10, NaH_2_PO_4_ 0.33, and glucose 5.5; pH 7.4 adjusted with 3M NaOH) (Bébarová et al., 2017). The addition of increasing concentrations of aminophylline (10 µM, 100 µM, 1 mM, and 10 mM; Fagron) followed. Each concentration was prepared in Tyrode’s solution from 1 mM stock solution of aminophylline. The MCG was recorded for further analysis; each measurement point consisted of 10 min of stabilization time, followed by 10 min of measurement. Control experiments were conducted in the same setting without aminophylline in the treatments.

The contraction of beating EBs was recorded as a force value in time (MCG). In-house built MATLAB-based script located R and S peaks for each contractive event and their respective vertical deflection and time values. From these values, R-R (sec), R-S (contraction force; nN), and BR (beat rate; beats per minute) were calculated, averaged, and normalized to the respective baseline values recorded in Tyrode’s solution.

### Multielectrode Array Measurements

The field potential of the HL-1 cells was measured using MEA2100-mini-60 (Multi Channel Systems). Drug responses were measured after initial equilibration in Claycomb medium, followed by the increasing concentrations of aminophylline in Claycomb medium (8, 16, 32, 64, 128, 256, and 512 µM). Measurement points consisted of 3 min of stabilization time, followed by 5 min of measurement. As a monolayer, HL-1 cells are more sensitive to a treatment than hPSC-CM clusters. Therefore, treatment measurement times and concentration ranges were modified.

MEA recordings were analyzed using Multi-Channel Analyzer software and in-house Python script, which processed the data in a similar way; however, the resulting parameters were only R-R (sec) and BR (beat rate; beats per minute). R-S (amplitude; µV) values in case of MEA recordings do not correlate with R-S values measured *via* AFM; therefore, they were not used in this study. Noisy and non-representative MEA channels were eliminated, and at least 3 channels were then used for calculations.

### Measurements of Intracellular Cytosolic Ca^2+^

HL-1 cells were passaged and seeded onto an imaging dish with a polymer coverslip bottom (Ibidi) and allowed to adhere. Fluo-8 (490/525; AAT Bioquest) was added into the medium (final concentration 2 µM; stock solution in DMSO 2 mM), and the cells were incubated for 20 min at 37°C. After the incubation, the solution was replaced with fresh medium, and the cells were placed on the heated stage (37°C) of an inverted microscope. Ca^2+^ images in the line-scan (line size 3.37 µm, 100 Hz) mode were recorded using a laser scanning confocal microscope Olympus FL1200 (Olympus) with a ×40 water immersion objective, in the x–y mode. Kinetic properties of intracellular Ca^2+^ were analyzed *via* the in-house Python-based script (Kabanov, 2021).

### Measurement of HL-1 Viability

The HL-1 cells were passaged and mixed with PBS containing aminophylline (final concentration 512 µM) or only PBS for control. The cells were incubated for 8 min after which their viability was analyzed via the trypan blue dye exclusion method (Vicell-XR, Beckman Coulter).

### Statistical Analyses

Statistical evaluation was carried out with the use of GraphPad Prism 8.5 software (GraphPad Software). All data were tested for outliers by the ROUT (Q = 1%) method, and available normality tests were performed for the obtained data. In case of hPSC-CM measurements, Brown–Forsythe ANOVA with Games–Howell multiple comparisons test was used to assess the statistical significance of the differences on normally distributed group pairs. In the case of arrhythmia analysis, R-R for each contractive event was extracted *via* processing scripts as mentioned before. R-R values over 1 or 3 s (cut-off) in HL-1 and hPSC-CMs, respectively, were quantified in all treatment groups and compared to controls. Choice of the cut-off value for hPSC-CMs was based on clinical experience with arrhythmia in human patients. The cut-off value for HL-1 was reduced with respect to the fact that HL-1 is a murine cell line with a higher overall beat rate. The resulting contingency tables were statistically evaluated by using the chi-square test with Yates’s correction. The use of the individual statistical test is specified in appropriate figure legends and in Supplementary Data. Additionally, datasets containing complete raw data from all measurements are available at the online open-access repository (Klimovic et al., 2021).

## Results

### Positive Chronotropic and Inotropic Effects of Aminophylline on hPSC-CMs

In the case of hPSC-CMs, two groups of treatment concentrations were determined according to the literature (Aslaksen et al., 1981; Goldberg et al., 1986; Rowe et al., 1988; Higgins et al., 1995; Shannon, 1999). The “therapeutic concentration/overdose” group consisted of 10 µM, 100 µM, and 1 mM of aminophylline treatments, while the 10-mM aminophylline treatment was considered “severe overdose” concentration. Consistent presence of troponin-positive cardiomyocytes in whole and dissociated embryoid bodies (EBs) was confirmed by immunostaining (representative example shown in Figure 2).

**FIGURE 3:**
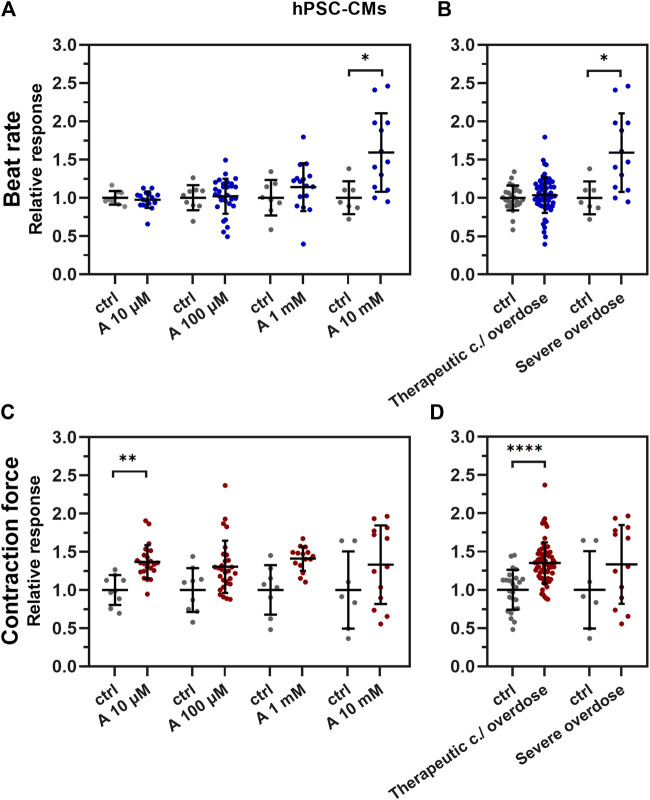
Effect of aminophylline (A) on contractile properties of 3D cardiac clusters. Scatterplots (mean ± standard deviation) of the BR (blue) and contraction force (red) overall changes, normalized to a baseline measurement (relative response) and to control measurement means (*n* = 19 for A 10 µM, *n* = 30 for A 100 µM, *n* = 16 for A 1 mM, *n* = 13 for A 10 mM, *n* = 9 for BR ctrl and *n* = 7 for contraction force ctrl). At least four biological repetitions were used in each column. **(A)** BR of the measured EBs with 10 mM aminophylline (A 10 mM) was significantly increased compared to that of the control (*p* < 0.05). **(B)** Similarly, the BR of EBs treated with 10 mM concentration of aminophylline was significantly increased over that of the controls (*p* < 0.05). **(C)** Contraction force of EBs with 10 µM aminophylline treatment was significantly increased over that of the control (*p* < 0.05), with a similar trend in higher concentrations. **(D)** Group analyses then showed a strong statistically significant effect of therapeutic concentration / overdose (10 μM and 100 μM and 1 mM) aminophylline over the controls. (Brown–Forsythe and Welch ANOVA tests were used in all analyses).

The positive chronotropic effect of aminophylline on hPSC-CMs was seen only in the 10 mM concentration of aminophylline (Figures 3A,B; *p* < 0.05; the remaining *p*-values are given in Supplementary Table S1 and Supplementary Table S2). The positive inotropic effect was evident by a significantly increased contraction force of EBs in lower concentrations of aminophylline that was in contrast to its chronotropic effect (Figure 3D; *p* < 0.0001; the remaining *p*-values are given in Supplementary Table S3). Statistical analysis showed that this effect was significant in 10 µM concentration, with a similar trend in the concentrations of 100 µM and 1 mM (Figure 3C; *p* < 0.01; the remaining *p*-values are given in Supplementary Table S4). Linear regression analysis showed that the beat rate increase correlates with the concentration of aminophylline (Supplementary Figure S2A,B, *p* < 0.0001). On the contrary, this relationship was not proven in case of contraction force (Supplementary Figure S2A,B). To further strengthen the presented results, the washout experiment was performed on hPSC-CMs. The results showed that the BR of EBs increases significantly after administration of aminophylline; however, it decreased again during the washout period, suggesting that the chronotropic and inotropic effects is likely due to the effect of aminophylline and not due to irreversible cellular damage (Supplementary Figure S3).

In order to further explain molecular mechanisms, hPSC-CM cells were treated with 1 µM adenosine, followed by a combination of 1 µM adenosine and 1 mM aminophylline, a well-known non-selective adenosine receptor antagonist. The results showed an insignificant trend toward the adenosine-antagonizing aminophylline effect (Supplementary Figure S3B).

### Positive Chronotropic Effect of Aminophylline on HL-1

To test whether chronotropic effects of aminophylline are model-independent, field potential measurements of the HL-1 cardiac cell line treated with aminophylline were conducted. The concentrations of aminophylline in a range of 8 up to 512 µM were used. Higher concentrations turned out to be toxic for the cells, which can be explained by higher treatment efficacy on the cellular monolayer of HL-1, as opposed to the cellular cluster of hPSC-CMs. Non-toxicity of selected concentrations was experimentally tested by trypan blue staining **(** Supplementary Figure S3C).

**FIGURE 4:**
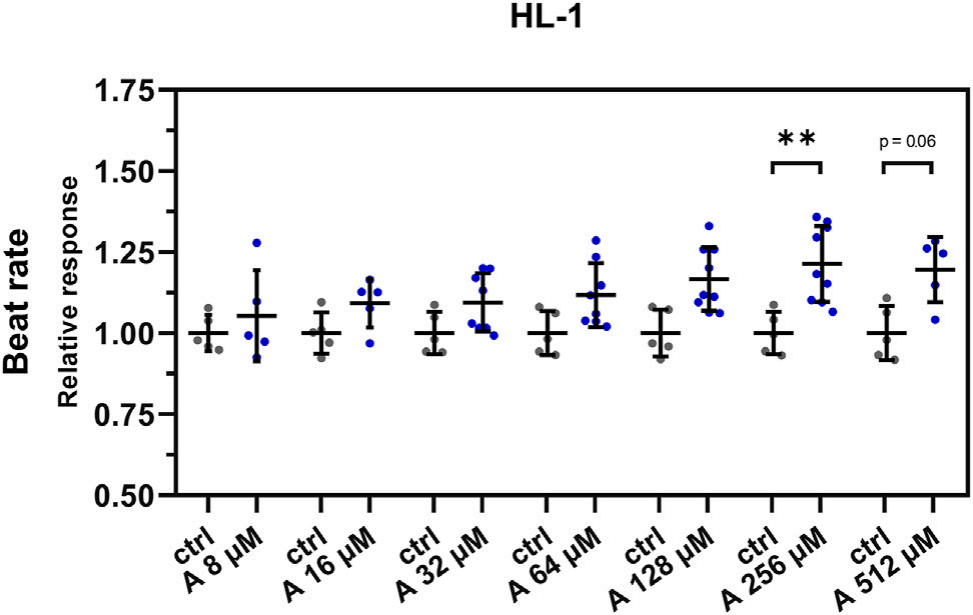
Effect of aminophylline (A) on the BR of HL-1 cells. Scatterplots (mean ± standard deviation) of the BR (blue) normalized to a baseline measurement (relative response) and to control measurement means (*n* = 5 for A 8 and 16 µM, *n* = 8 for 32 and 64 µM, *n* = 9 for 128 and 256 µM, *n* = 5 for 512 µM and *n* = 5 for ctrl). At least three biological repetitions were used in each column. Results showed linear increase of BR correlating with a higher concentration of aminophylline, with statistically significant and partially significant changes of BR 256 and 512 µM measurements compared that of the control (A 256 vs. ctlr *p* < 0.01, A 526 vs. ctlr *p* = 0.06, ordinary one-way ANOVA).

The results of these experiments showed a similar positive chronotropic effect with increasing concentration of aminophylline. Compared to controls, the BR was significantly increased only in the cells treated with 256 µM aminophylline; however, we observed a similar non-significant trend also in the case of the 512-µM treatment (Figure 4; 256 µM aminophylline vs. the control *p* < 0.05; 512 µM aminophylline vs. the control *p* < 0.06; the remaining *p*-values are given in Supplementary Table S5).

### Exposure to Aminophylline Causes Spontaneous Ca^2+^ Releases Leading to Arrhythmia

**FIGURE 5:**
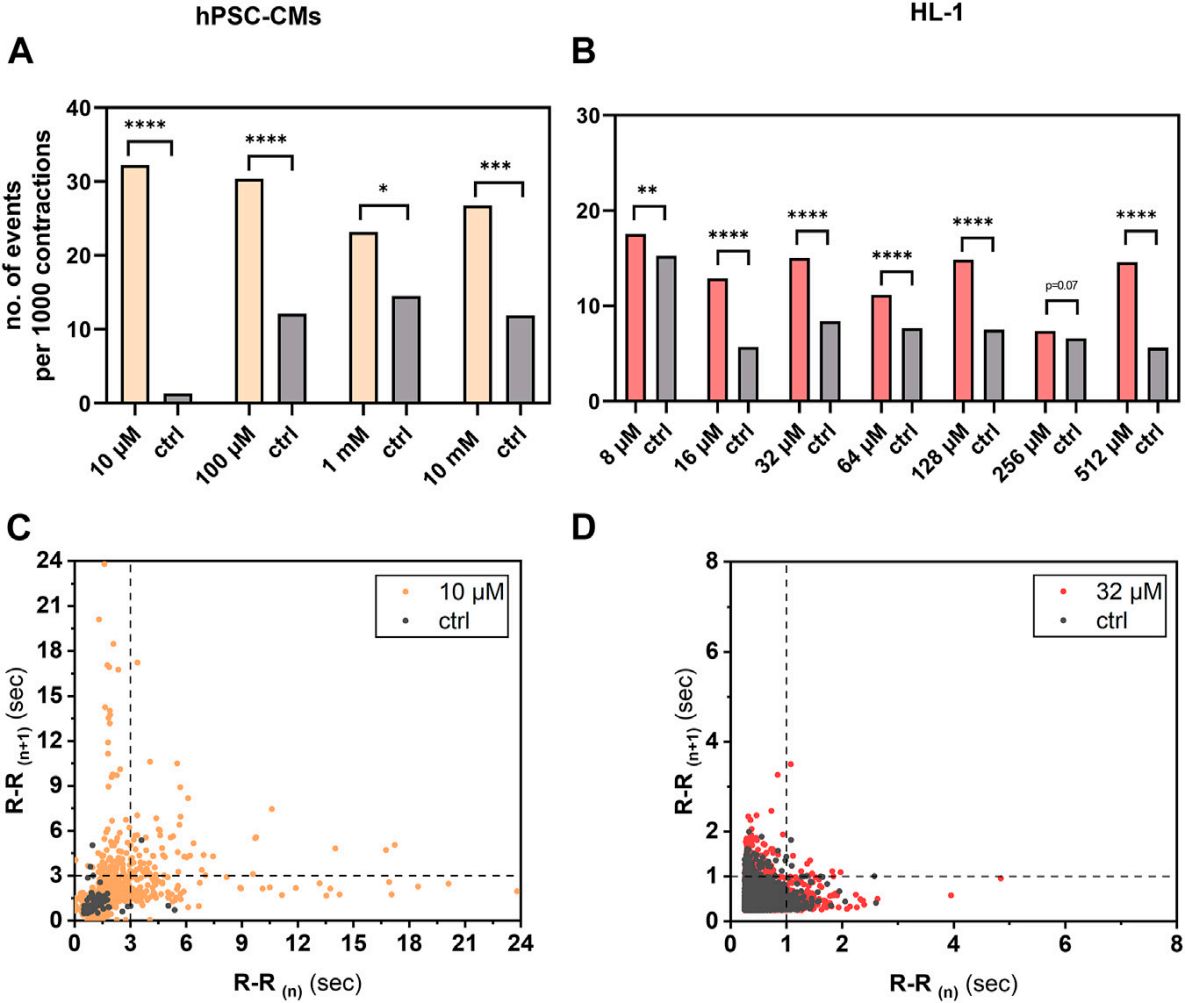
Analysis of arrhythmogenic effect of aminophylline on hPSC-CMs and HL-1 cells. R-R values over 3 s in case of hPSC-CM measurements or 1 s in case of HL-1 of aminophylline and control measurements were subtracted, and the resulting contingency tables were statistically analyzed. Column graphs show sums of cutoff R-R values (arrhythmic events) per 1,000 contractions in each treatment group and controls of **(A)** hPSC-CMs and **(B)** HL-1 experiments (chi-square test with Yates’ correction). Results show significant or partially significant higher frequency of aminophylline group cutoff values compared to that of the control. Poincaré plots of R-R values of representative concentration for **(C)** hPSC-CMs and **(D)** HL-1 experiments with visible cutoff lines.

Among the adverse effects of aminophylline apparent in clinical practice, arrhythmias are most frequent. To test whether this effect is also present in our *in vitro* hPSC-CM model and HL-1 cardiomyocytes, we assessed the number of R-R intervals (inter-beat interval length) over 3 s or 1 s, respectively. Our results showed that the frequency of cutoff R-R values in measurements with aminophylline was significantly higher than that in controls in both models (Figure 5; *p*-values in Supplementary Table S6 and Supplementary Table S7 **)**.

**FIGURE 6:**
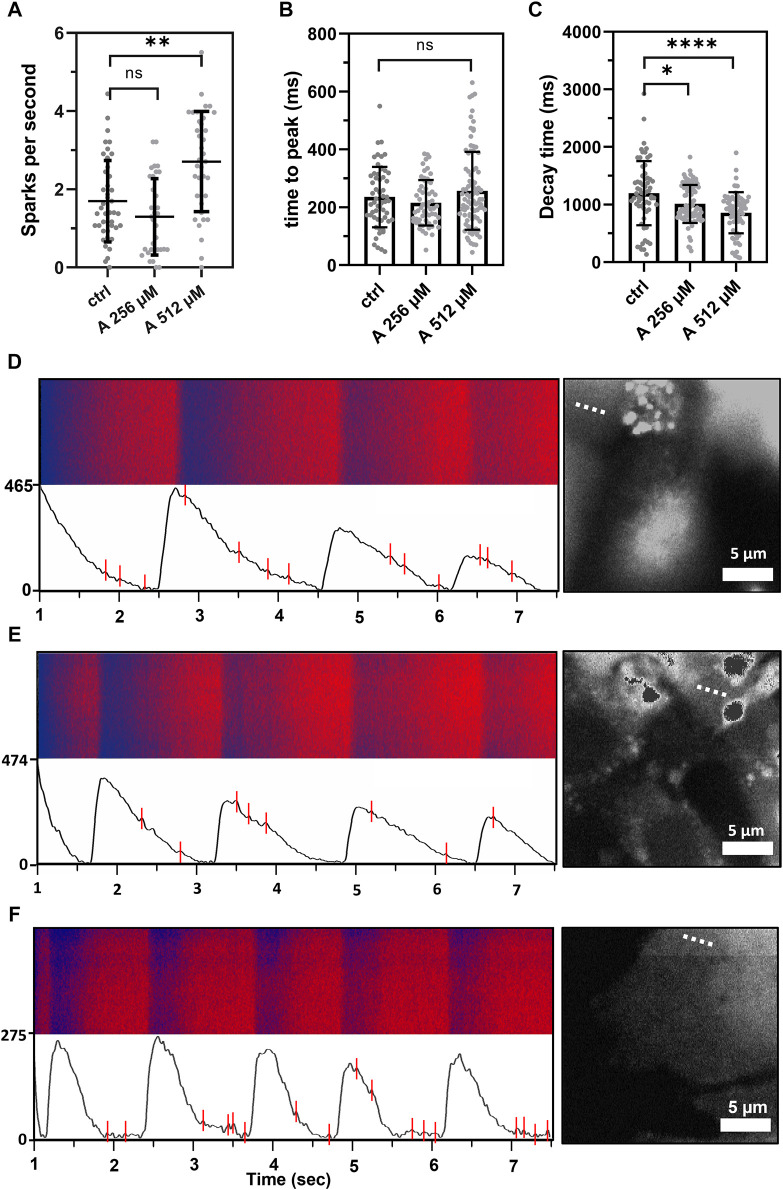
Calcium sparks measured as extra events in the fluorescence in-time signal, showing leakage of calcium from the sarcoplasmic reticulum. **(A)** Scatterplot (mean ± standard deviation) showing calcium leakage events in a one-second period in control cells compared to the treatment by 256 and 512 μM aminophylline (A; *n* = 43 for ctlr, *n* = 33 for 256 µM and *n* = 37 for 512 µM). The frequency of calcium leakage events of cells treated with 512 µM aminophylline was significantly higher than that of the control (A 512 vs. ctlr *p* < 0.01, ordinary one-way ANOVA). **(B,C)** Scatterplots (mean ± standard deviation) showing times to peak and decay times of the control compared to the treatments. Aminophylline caused no significant change to time to peak compared to the control; however, decay time significantly decreased in a linear trend (*n* = 65 for ctrl, *n* = 89 for A 256 µM, *n* = 77 for A 512 µM; ctlr vs. A 256 µM *p* < 0.05, ctlr vs. A 512 µM *p* < 00.001; Kruskal–Wallis test). **(D–F)** Fluorescence line profiles were measured in time, typical recordings together with fluorescence–time curves were filtered for the presence of noise, and calcium sparks were found—labeled as red lines (B = control, C = 256 µM, D = 512 µM). Each recording is accompanied with a fluorescence image showing the exact location of line scan on the cell (dashed line).

To further investigate this effect, intracellular cytosolic Ca^2+^ events were measured on HL-1 cells in the presence of 256 and 512 µM aminophylline. A significantly higher number of Ca^2+^ sparks was detected in the presence of 512 µM aminophylline than the control (Figure 6 **,** 512 µM aminophylline vs. the control *p* < 0.0001). A similar effect was not detected in the presence of 256 µM aminophylline. Higher concentrations of aminophylline cause arrhythmic events presented by calcium leakage events (sparks). This is in good agreement with the measurement of electrical activity of cells (Figure 5B), where the concentration of 256 µM did not cause a significantly higher occurrence of arrhythmia; however, the concentration of 512 µM leads to rhythm irregularities. Last, the time to peak and decay time of main calcium waves were analyzed. While aminophylline caused no change in time to peak, the decay time significantly decreased, corresponding to an increased BR (Figures 6B,C).

## Discussion

This is the first study demonstrating the proarrhythmic action of aminophylline on human cardiomyocytes *in vitro*. To monitor aminophylline’s effect at a wide range of concentrations, AFM was used on cell clusters [embryoid bodies (EBs)] formed by aggregation of human pluripotent stem cell–derived cardiomyocytes. Furthermore, the effect of aminophylline was investigated *via* a multielectrode array and by means of intracellular cytosolic Ca^2+^ measurement on HL-1 cells. Despite the differences between the models such as monolayer growth, cellular populations, or the fact that it is a murine cell type, HL-1 cells are considered a standard drug screening platform; therefore, we chose them to confirm our findings.

A missing link between the previous experimental data and aminophylline-induced arrhythmias observed in clinical practice was identified. Our most important finding was that aminophylline had two principal actions of arrhythmogenicity, the first being concentration-dependent (“deterministic”) and the second one concentration-independent (“stochastic”). First, we observed a linear concentration-dependent positive chronotropic effect with increasing aminophylline concentrations in hPSC-CMs (Figures 3A,B and Supplementary Figure S2A,B). Moreover, this effect was also confirmed to an extent on the HL-1 cellular line (Figure 4). This may be related to previous clinical findings that demonstrated that theophylline’s higher plasmatic levels were associated with an increased heart rate (sinus tachycardia); the relationship was linear and concentration-dependent (Vestal et al., 1983; Nadkarni et al., 1988). This effect is sometimes used to treat severe, atropine-resistant bradycardia (Pasnoori and Leesar, 2004) or other indications (Conte et al., 2017). The inotropic effect was non-linear in our study. An increased inotropic effect was present only in lower aminophylline concentrations. This response may be attributed to reaching peak-shortening amplitude, as a physical parameter of cells and cluster, respectively, and partial immaturity may be responsible for lower number of present sarcomeres and, thus, lower shortening capability of the cells (Fraticelli et al., 1989).

In parallel to the “deterministic” effect, we observed a second arrhythmogenic action of aminophylline expressed as a concentration-independent occurrence of RR interval abnormities. This action was stochastic, independent of the applied aminophylline concentration. It consisted of repeated tachycardia-like periods followed by bradycardia-like periods (not illustrated), which is reflected by the extreme R-R intervals in Figure 5 that occurred significantly more often in the cells exposed to aminophylline. Even though certain beat rate variability was present in spontaneously beating hPSC-derived CMs in previous studies (Mandel et al., 2012; Binah et al., 2013; Niehoff et al., 2019), similar events have never been previously described to our best knowledge. We speculate that this effect may be an experimental equivalent to the sick sinus syndrome (Tse et al., 2017) or to tachy–brady alternating episodes in patients with AF, an arrhythmia that was previously observed both in aminophylline overdose cases and at therapeutic levels of the drug (Laaban et al., 1988; Bittar and Friedman, 1991; Varriale and Ramaprasad, 1993).

Attempts to reveal mechanisms of the arrhythmogenic action of aminophylline/theophylline have been performed previously, using various animal models. In rats, aminophylline induced tachycardia and elevated blood pressure and potential cardiac ischemia (Shamsuzzaman et al., 2016), which may be related to theophylline-induced myocardial fibrosis found as a long-term concentration-/dose-dependent side effect in a notable portion of rats (Onodera et al., 2001). In a canine model, aminophylline significantly enhanced AV nodal and His–Purkinje conduction; this effect was potentiated in combined therapy with metaproterenol (Komadina et al., 1992).

Studies published so far have not provided a clear clue to the genesis of proarrhythmic effects of aminophylline/theophylline, namely, AF. Aminophylline effects (likely an equivalent of the sinus tachycardia in patients) may be explained by the effect of elevated cAMP and the absence of the inhibitory effect of adenosine (Belardinelli et al., 1995) on cardiac ionic currents (see insignificant trend in Supplementary Figure S3B).

As studied in detail by Kong et al. (2008), caffeine (another type of methylxanthine), aminophylline, and theophylline preferentially potentiated luminal Ca^2+^ activation of the ryanodine type 2 (RyR2) receptor, reduced the threshold for spontaneous Ca^2+^ release, and increased the basal activity, inducing repeated quantal Ca^2+^ releases. As is well-known, the accumulation of Ca^2+^ in the cytoplasm may result in delayed afterdepolarizations, which can trigger arrhythmia. Considering the increased occurrence of Ca^2+^ sparks in HL-1 cardiomyocytes treated with 512 µM aminophylline (Figure 6), the concentration-independent “stochastic” effect of aminophylline observed in our study may be a consequence of microdomain cAMP level–related diastolic release of Ca^2+^ *via* independent clusters of RyR2 receptors (Berisha et al., 2021). Such transient Ca^2+^ accumulation may induce an increased BR and, following Ca^2+^ depletion, results in a pause in the investigated cardiac cell’s beating clusters. Such chain of events might also be amplified by the local cAMP level–induced alteration of the current amplitude *via* modulation of hERG potassium channel phosphorylation (Cui et al., 2000). Detailed subcellular mechanisms of this effect should be studied in the future.

To our best knowledge, this is the first study reporting results of experiments with aminophylline conducted on human cardiomyocytes derived from hPSCs. As such, this set of experiments showed that our comprehensive monitoring system is a unique and valuable tool for cardiotoxicity vs. safety testing of various molecules or drugs. Our results may present the missing link between the described subcellular mechanisms of methylxanthine arrhythmogenesis and the clinical variability of arrhythmic events related to the theophylline treatment in clinical practice.

## Conclusion/Summary

We conclude that aminophylline had two parallel arrhythmogenic mechanisms of action on EBs: a concentration-dependent (“deterministic”) effect, presenting with an increased beat rate (potential clinical correlate: sinus tachycardia), and a concentration-independent (“stochastic”) effect, which was characterized by tachycardia-like episodes alternating with long pauses (potential clinical correlate: atrial fibrillation). Our comprehensive monitoring system is a unique and valuable tool for cardiotoxicity vs. safety testing of various molecules or drugs.

## Study Limitations

The study presents the effect of aminophylline on contractile properties of human hPSC-CMs along with results of field potential measurements on the HL-1 cardiac cell line that further confirmed the effects. First, it has to be considered that hPSC-derived CMs represented immature/neonatal phenotype possibly affecting drug effect kinetics [e.g., altered IK1 levels which may affect resting potential, repolarization, and depolarization dynamics (Sartiani et al., 2007)]. Second, if different hPSC lines are used to generate embryoid bodies, it is likely that there will be different degrees of maturity and the effect of aminophylline might also differ. The last limitation is that field potential measurements can generate information about the BR but not about contraction force; therefore, the results with HL-1 cells confirmed only the chronotropic effect of aminophylline.

## Data Availability

The original contributions presented in the study are included in the article/Supplementary Material; further inquiries can be directed to the corresponding author.

## Abbreviations

sAFM, atomic force microscope; bpm, beats per minute; CBBs, cell-based biosensors; CCTL, center for cell therapy line; CM, cardiomyocyte; EB, embryoid body; ECC, excitation–contraction coupling; hESC, human embryonic stem cell; hiPSC, human-induced pluripotent stem cell; hPSC, human pluripotent stem cell (hESC and hiPSC); hPSC-CMs, pluripotent stem cell–derived cardiomyocytes.

## References

[B1] Acimovic I. Refaat M. M. Moreau A. Salykin A. Reiken S. Sleiman Y. (2018). Post-translational Modifications and Diastolic Calcium Leak Associated to the Novel RyR2-D3638a Mutation Lead to CPVT in Patient-specific hiPSC-Derived Cardiomyocytes. J. Clin. Med. 7, 423. 10.3390/jcm7110423 PMC6262462 30413023

[B3] Aslaksen A. Bakke O. M. Vigander T. (1981). Comparative Pharmacokinetics of Theophylline and Aminophylline in Man. Br. J. Clin. Pharmacol. 11, 269–273. 10.1111/j.1365-2125.1981.tb00533.x 7213528 PMC1401615

[B4] Bébarová M. Matejovič P. Švecová O. Kula R. Šimurdová M. Šimurda J. (2017). Nicotine at Clinically Relevant Concentrations Affects Atrial Inward Rectifier Potassium Current Sensitive to Acetylcholine. Naunyn-schmiedeberg's Arch. Pharmacol. 390, 471–481. 10.1007/s00210-017-1341-z 28160016

[B5] Belardinelli L. Shryock J. C. Song Y. Wang D. Srinivas M. (1995). Ionic Basis of the Electrophysiological Actions of Adenosine on Cardiomyocytes. FASEB J. 9, 359–365. 10.1096/fasebj.9.5.7896004 7896004

[B6] Berisha F. Götz K. R. Wegener J. W. Brandenburg S. Subramanian H. Molina C. E. (2021). cAMP Imaging at Ryanodine Receptors Reveals β2-Adrenoceptor Driven Arrhythmias. Circ. Res. 129, 81–94. 10.1161/CIRCRESAHA.120.318234 33902292

[B7] Binah O. Weissman A. Itskovitz-Eldor J. Rosen M. R. (2013). Integrating Beat Rate Variability: from Single Cells to Hearts. Heart Rhythm 10, 928–932. 10.1016/j.hrthm.2013.02.013 23416376 PMC3923529

[B8] Bittar G. Friedman H. S. (1991). The Arrhythmogenicity of Theophylline. A Multivariate Analysis of Clinical Determinants. Chest 99, 1415–1420. 10.1378/chest.99.6.1415 2036824

[B9] Burridge P. W. Keller G. Gold J. D. Wu J. C. (2012). Production of De Novo Cardiomyocytes: Human Pluripotent Stem Cell Differentiation and Direct Reprogramming. Cell Stem Cell 10, 16–28. 10.1016/j.stem.2011.12.013 22226352 PMC3255078

[B10] Caluori G. Pribyl J. Cmiel V. Pesl M. Potocnak T. Provaznik I. (2019a). Simultaneous Study of Mechanobiology and Calcium Dynamics on hESC-Derived Cardiomyocytes Clusters. J. Mol. Recognit. 32, e2760. 10.1002/jmr.2760 30084213

[B11] Caluori G. Pribyl J. Pesl M. Jelinkova S. Rotrekl V. Skladal P. (2019b). Non-invasive Electromechanical Cell-Based Biosensors for Improved Investigation of 3D Cardiac Models. Biosens. Bioelectron. 124-125 (125), 129–135. 10.1016/j.bios.2018.10.021 30366257

[B12] Charytan D. Jansen K. (2003). Severe Metabolic Complications from Theophylline Intoxication. Nephrology (Carlton) 8, 239–242. 10.1046/j.1440-1797.2003.00181.x 15012710

[B13] Conte L. Pugliese N. R. Giannoni A. (2017). Reversal of Ticagrelor-Induced Arrhythmias and Cheyne-Stokes Respiration with Aminophylline Infusion. J. Cardiovasc. Pharmacol. 70, 290–292. 10.1097/FJC.0000000000000518 28704306

[B14] Cui J. Melman Y. Palma E. Fishman G. I. McDonald T. V. (2000). Cyclic AMP Regulates the HERG K(+) Channel by Dual Pathways. Curr. Biol. 10, 671–674. 10.1016/s0960-9822(00)00516-9 10837251

[B15] Kabanov D. (2021). DaniilKabanov/CardioScripts. Available at: https://github.com/DaniilKabanov/CardioScripts [Accessed September 14, 2021].

[B16] Dinarelli S. Girasole M. Spitalieri P. Talarico R. V. Murdocca M. Botta A. (2018). AFM Nano-Mechanical Study of the Beating Profile of hiPSC-Derived Cardiomyocytes Beating Bodies WT and DM1. J. Mol. Recognit 31, e2725. 10.1002/jmr.2725 29748973

[B17] Dvorak P. Dvorakova D. Koskova S. Vodinska M. Najvirtova M. Krekac D. (2005). Expression and Potential Role of Fibroblast Growth Factor 2 and its Receptors in Human Embryonic Stem Cells. Stem Cells 23, 1200–1211. 10.1634/stemcells.2004-0303 15955829

[B18] Ellis E. F. (1985). Theophylline Toxicity. J. Allergy Clin. Immunol. 76, 297–301. 10.1016/0091-6749(85)90645-1 4019958

[B19] Fraticelli A. Josephson R. Danziger R. Lakatta E. Spurgeon H. (1989). Morphological and Contractile Characteristics of Rat Cardiac Myocytes from Maturation to Senescence. Am. J. Physiol. 257, H259–H265. 10.1152/ajpheart.1989.257.1.H259 2750941

[B20] Goldberg M. J. Park G. D. Berlinger W. G. (1986). Treatment of Theophylline Intoxication. J. Allergy Clin. Immunol. 78, 811–817. 10.1016/0091-6749(86)90066-7 3534061

[B21] Goudis C. A. (2017). Chronic Obstructive Pulmonary Disease and Atrial Fibrillation: An Unknown Relationship. J. Cardiol. 69, 699–705. 10.1016/j.jjcc.2016.12.013 28188041

[B22] Higgins R. M. Hearing S. Goldsmith D. J. Keevil B. Venning M. C. Ackrill P. (1995). Severe Theophylline Poisoning: Charcoal Haemoperfusion or Haemodialysis. Postgrad. Med. J. 71, 224–226. 10.1136/pgmj.71.834.224 7784283 PMC2398059

[B23] Hoppe L. K. Muhlack D. C. Koenig W. Carr P. R. Brenner H. Schöttker B. (2018). Association of Abnormal Serum Potassium Levels with Arrhythmias and Cardiovascular Mortality: a Systematic Review and Meta-Analysis of Observational Studies. Cardiovasc. Drugs Ther. 32, 197–212. 10.1007/s10557-018-6783-0 29679302

[B24] Horita N. Miyazawa N. Kojima R. Inoue M. Ishigatsubo Y. Kaneko T. (2016). Chronic Use of Theophylline and Mortality in Chronic Obstructive Pulmonary Disease: A Meta-Analysis. Arch. Bronconeumol 52, 233–238. 10.1016/j.arbres.2015.02.021 26612542

[B25] Huerta C. Lanes S. F. García Rodríguez L. A. (2005). Respiratory Medications and the Risk of Cardiac Arrhythmias. Epidemiology 16, 360–366. 10.1097/01.ede.0000158743.90664.a7 15824553

[B2] International Stem Cell Initiative Adewumi O. Adewumi O. Aflatoonian B. Ahrlund-Richter L. Amit M. Andrews P. W. (2007). Characterization of Human Embryonic Stem Cell Lines by the International Stem Cell Initiative. Nat. Biotechnol. 25, 803–816. 10.1038/nbt1318 17572666

[B26] Jelinkova S. Fojtik P. Kohutova A. Vilotic A. Marková L. Pesl M. (2019). Dystrophin Deficiency Leads to Genomic Instability in Human Pluripotent Stem Cells via NO Synthase-Induced Oxidative Stress. Cells 8. 10.3390/cells8010053 PMC6356905 30650618

[B27] Jelinkova S. Vilotic A. Pribyl J. Aimond F. Salykin A. Acimovic I. (2020). DMD Pluripotent Stem Cell Derived Cardiac Cells Recapitulate *In Vitro* Human Cardiac Pathophysiology. Front. Bioeng. Biotechnol. 8, 535. 10.3389/fbioe.2020.00535 32656189 PMC7325914

[B28] Klimovic S. Scurek M. Pesl M. Beckerova D. Jelinkova S. Urban T. (2021). Aminophylline Induces Two Types of Arrhythmic Events in Human Pluripotent Stem Cell-Derived Cardiomyocytes - Dataset. 10.5281/zenodo.4552607 PMC8802108 35111056

[B29] Komadina K. H. Carlson T. A. Strollo P. J. Navratil D. L. (1992). Electrophysiologic Study of the Effects of Aminophylline and Metaproterenol on Canine Myocardium. Chest 101, 232–238. 10.1378/chest.101.1.232 1345901

[B30] Krutá M. Šeneklová M. Raška J. Salykin A. Zerzánková L. Pešl M. (2014). Mutation Frequency Dynamics inHPRTLocus in Culture-Adapted Human Embryonic Stem Cells and Induced Pluripotent Stem Cells Correspond to Their Differentiated Counterparts. Stem Cell Dev. 23, 2443–2454. 10.1089/scd.2013.0611 PMC4186764 24836366

[B31] Laaban J. P. Iung B. Chauvet J. P. Psychoyos I. Proteau J. Rochemaure J. (1988). Cardiac Arrhythmias during the Combined Use of Intravenous Aminophylline and Terbutaline in Status Asthmaticus. Chest 94, 496–502. 10.1378/chest.94.3.496 3409727

[B32] Lee T. A. Schumock G. T. Bartle B. Pickard A. S. (2009). Mortality Risk in Patients Receiving Drug Regimens with Theophylline for Chronic Obstructive Pulmonary Disease. Pharmacotherapy 29, 1039–1053. 10.1592/phco.29.9.1039 19698009

[B33] Liu J. Sun N. Bruce M. A. Wu J. C. Butte M. J. (2012). Atomic Force Mechanobiology of Pluripotent Stem Cell-Derived Cardiomyocytes. PLoS ONE 7, e37559. 10.1371/journal.pone.0037559 22624048 PMC3356329

[B34] Mandel Y. Weissman A. Schick R. Barad L. Novak A. Meiry G. (2012). Human Embryonic and Induced Pluripotent Stem Cell-Derived Cardiomyocytes Exhibit Beat Rate Variability and Power-Law Behavior. Circulation 125, 883–893. 10.1161/CIRCULATIONAHA.111.045146 22261196 PMC3697086

[B35] Nadkarni S. Hay A. W. Faye S. Congdon P. J. (1988). The Relationship between Theophylline, Caffeine and Heart Rate in Neonates. Ann. Clin. Biochem. 25 ( Pt 4) (Pt 4), 408–410. 10.1177/000456328802500415 3214124

[B36] Nair P. Milan S. J. Rowe B. H. (2012). Addition of Intravenous Aminophylline to Inhaled Beta(2)-Agonists in Adults with Acute Asthma. Cochrane Database Syst. Rev. 12, CD002742. 10.1002/14651858.CD002742.pub2 23235591 PMC7093892

[B37] Niehoff J. Matzkies M. Nguemo F. Hescheler J. Reppel M. (2019). The Effect of Antiarrhythmic Drugs on the Beat Rate Variability of Human Embryonic and Human Induced Pluripotent Stem Cell Derived Cardiomyocytes. Sci. Rep. 9, 14106. 10.1038/s41598-019-50557-7 31575920 PMC6773847

[B38] Odstrcilik J. Cmiel V. Kolar R. Ronzhina M. Baiazitova L. Pesl M. (2015). “Computer Analysis of Isolated Cardiomyocyte Contraction Process via Advanced Image Processing Techniques,” in 2015 Computing in Cardiology Conference (CinC) (Nice, France: IEEE), 453–456. 10.1109/CIC.2015.7408684

[B39] Onodera K. Shibata M. Kojima J. Wachi M. Sogawa N. Furuta H. (2001). Toxicity of Theophylline Depends on Plasma Concentration by Single and Also Repeated Dosing in Rats. Pharmacol. Res. 44, 81–87. 10.1006/phrs.2001.0831 11516255

[B40] Pasnoori V. R. Leesar M. A. (2004). Use of Aminophylline in the Treatment of Severe Symptomatic Bradycardia Resistant to Atropine. Cardiol. Rev. 12, 65–68. 10.1097/01.crd.0000096418.72821.fa 14766019

[B41] Pesl M. Acimovic I. Pribyl J. Hezova R. Vilotic A. Fauconnier J. (2014). Forced Aggregation and Defined Factors Allow Highly Uniform-Sized Embryoid Bodies and Functional Cardiomyocytes from Human Embryonic and Induced Pluripotent Stem Cells. Heart Vessels 29, 834–846. 10.1007/s00380-013-0436-9 24258387

[B42] Pribyl J. Pešl M. Caluori G. Acimovic I. Jelinkova S. Dvorak P. (20191886). Biomechanical Characterization of Human Pluripotent Stem Cell-Derived Cardiomyocytes by Use of Atomic Force Microscopy. Methods Mol. Biol. 1886, 343–353. 10.1007/978-1-4939-8894-5_20 30374878

[B43] Rowe D. J. Watson I. D. Williams J. Berry D. J. (1988). The Clinical Use and Measurement of Theophylline. Ann. Clin. Biochem. 25 ( Pt 1), 4–26. 10.1177/000456328802500102 3281555

[B44] Sartiani L. Bettiol E. Stillitano F. Mugelli A. Cerbai E. Jaconi M. E. (2007). Developmental Changes in Cardiomyocytes Differentiated from Human Embryonic Stem Cells: a Molecular and Electrophysiological Approach. Stem Cells 25, 1136–1144. 10.1634/stemcells.2006-0466 17255522

[B45] Shamsuzzaman M. Kavita G. Arunabha R. (2016). Methylxanthine Induced Cardiotoxicity and its Mechanisms: An Experimental Study. MJMS 1, 10.

[B46] Shannon M. (1999). Life-threatening Events after Theophylline Overdose: a 10-year Prospective Analysis. Arch. Intern. Med. 159, 989–994. 10.1001/archinte.159.9.989 10326941

[B47] Singh D. Agusti A. Anzueto A. Barnes P. J. Bourbeau J. Celli B. R. (2019). Global Strategy for the Diagnosis, Management, and Prevention of Chronic Obstructive Lung Disease: the GOLD Science Committee Report 2019. Eur. Respir. J. 53. 10.1183/13993003.00164-2019 30846476

[B48] Souidi M. Sleiman Y. Acimovic I. Pribyl J. Charrabi A. Baecker V. (2021). Oxygen Is an Ambivalent Factor for the Differentiation of Human Pluripotent Stem Cells in Cardiac 2D Monolayer and 3D Cardiac Spheroids. Ijms 22, 662. 10.3390/ijms22020662 PMC7827232 33440843

[B49] Suissa S. Hemmelgarn B. Blais L. Ernst P. (1996). Bronchodilators and Acute Cardiac Death. Am. J. Respir. Crit. Care Med. 154, 1598–1602. 10.1164/ajrccm.154.6.8970341 8970341

[B50] Tse G. Liu T. Li K. H. Laxton V. Wong A. O. Chan Y. W. (2017). Tachycardia-bradycardia Syndrome: Electrophysiological Mechanisms and Future Therapeutic Approaches (Review). Int. J. Mol. Med. 39, 519–526. 10.3892/ijmm.2017.2877 28204831 PMC5360359

[B51] Ukena D. Schudt C. Sybrecht G. W. (1993). Adenosine Receptor-Blocking Xanthines as Inhibitors of Phosphodiesterase Isozymes. Biochem. Pharmacol. 45, 847–851. 10.1016/0006-2952(93)90168-v 7680859

[B52] Varriale P. Ramaprasad S. (1993). Aminophylline Induced Atrial Fibrillation. Pacing Clin. Electrophysiol. 16, 1953–1955. 10.1111/j.1540-8159.1993.tb00987.x 7694240

[B53] Vestal R. E. Eiriksson C. E. Musser B. Ozaki L. K. Halter J. B. (1983). Effect of Intravenous Aminophylline on Plasma Levels of Catecholamines and Related Cardiovascular and Metabolic Responses in Man. Circulation 67, 162–171. 10.1161/01.cir.67.1.162 6336606

[B54] Ye C. Miao C. Yu L. Dong Z. Zhang J. Mao Y. (2019). Factors Affecting the Efficacy and Safety of Aminophylline in Treatment of Apnea of Prematurity in Neonatal Intensive Care Unit. Pediatr. Neonatol 60, 43–49. 10.1016/j.pedneo.2018.03.008 29673564

[B55] Zatloukal J. Brat K. Neumannova K. Volakova E. Hejduk K. Kocova E. (2020). Chronic Obstructive Pulmonary Disease - Diagnosis and Management of Stable Disease; a Personalized Approach to Care, Using the Treatable Traits Concept Based on Clinical Phenotypes. Position Paper of the Czech Pneumological and Phthisiological Society. Biomed. Pap. Med. Fac. Univ. Palacky Olomouc Czech Repub 164, 325–356. 10.5507/bp.2020.056 33325455

