# Supplementary material for: Aminophylline Induces Two Types of Arrhythmic Events in Human Pluripotent Stem Cell–Derived Cardiomyocytes
Source: Front Pharmacol. 2022 Jan 17;12:789730. doi: 10.3389/fphar.2021.789730 (PMC8802108; doi:10.3389/fphar.2021.789730)
Supplement: Supplementary file 1 [file DataSheet1.docx]

Aminophylline induces two types of arrhythmic events in human pluripotent stem cell-derived cardiomyocytes.

Supplementary Data

| Games-Howell's multiple comparisons test | Mean Diff. | 95.00% CI of diff. | Significant? | Summary | Adjusted P Value |
| --- | --- | --- | --- | --- | --- |
| A 10 µM vs. ctrl | -0.02497 | -0.1554 to 0.1054 | No | ns | 0.9975 |
| A 100 µM vs. ctrl | 0.01922 | -0.2148 to 0.2533 | No | ns | >0.9999 |
| A 1 mM vs. ctrl | 0.1394 | -0.2468 to 0.5256 | No | ns | 0.9130 |
| A 10 mM vs. ctrl | 0.5908 | 0.03032 to 1.151 | Yes | * | 0.0348 |

**Table 1:** Statistical differences calculated for relative responses of beat rate of hPSC-CMs with aminophylline and control – concentration analysis.

**Table 2:** Statistical differences calculated for relative responses of beat rate of hPSC-CMs with aminophylline and control – group analysis.

| Games-Howell's multiple comparisons test | Mean Diff. | 95.00% CI of diff. | Significant? | Summary | Adjusted P Value |
| --- | --- | --- | --- | --- | --- |
| Clinically relevant vs. ctrl | 0.03588 | -0.07715 to 0.1489 | No | ns | 0.8368 |
| Overdose vs. ctrl | 0.5908 | 0.1268 to 1.055 | Yes | * | 0.0102 |

**Table 3:** Statistical differences calculated for relative responses of contraction force of hPSC-CMs with aminophylline and control – group analysis.

| Games-Howell's multiple comparisons test | Mean Diff. | 95.00% CI of diff. | Significant? | Summary | Adjusted P Value |
| --- | --- | --- | --- | --- | --- |
| Clinically relevant vs. ctrl | 0.3499 | 0.1880 to 0.5118 | Yes | **** | <0.0001 |
| Overdose vs. ctrl | 0.3312 | -0.3721 to 1.035 | No | ns | 0.5282 |

**Table 4:** Statistical differences calculated for relative responses of contraction force of hPSC-CMs with aminophylline and control – concentration analysis.

| Games-Howell's multiple comparisons test | Mean Diff. | 95.00% CI of diff. | Significant? | Summary | Adjusted P Value |
| --- | --- | --- | --- | --- | --- |
| A 10 µM vs. ctrl | 0.3690 | 0.09404 to 0.6440 | Yes | ** | 0.0050 |
| A 100 µM vs. ctrl | 0.3037 | -0.09399 to 0.7014 | No | ns | 0.2091 |
| A 1 mM vs. ctrl | 0.4098 | -0.05925 to 0.8789 | No | ns | 0.0971 |
| A 10 mM vs. ctrl | 0.3312 | -0.5251 to 1.187 | No | ns | 0.8470 |

**Table 5:** Statistical differences calculated for relative responses of HL1 cells treated with aminophylline versus controls.

| Tukey's multiple comparisons test | Mean Diff, | 95,00% CI of diff, | Significant? | Summary | Adjusted P Value |
| --- | --- | --- | --- | --- | --- |
| A 8 µM vs. ctrl | 0,05352 | -0,1472 to 0,2543 | No | ns | 0,9996 |
| A 16 µM vs. ctrl | 0,09254 | -0,1082 to 0,2933 | No | ns | 0,9399 |
| A 32 µM vs. ctrl | 0,09447 | -0,08649 to 0,2754 | No | ns | 0,8624 |
| A 64 µM vs. ctrl | 0,1172 | -0,06376 to 0,2982 | No | ns | 0,5956 |
| A 128 µM vs. ctrl | 0,1666 | -0,01041 to 0,3437 | No | ns | 0,0857 |
| A 256 µM vs. ctrl | 0,2134 | 0,03634 to 0,3904 | Yes | ** | 0,0057 |
| A 512 µM vs. ctrl | 0,1961 | -0,004671 to 0,3968 | No | ns | 0,0622 |

**Table 6:** Contingency statistical results of cutoff R-R values of aminophylline treated hPSC-CMs versus control.

| Chi-square with Yates' correction | Chi-square, df | z | P value | P value summary | One- or two-sided | Statistically significant (P < 0.05)? |
| --- | --- | --- | --- | --- | --- | --- |
| 10 µM vs. Ctrl | 88.60, 1 | 9.413 | <0.0001 | **** | Two-sided | Yes |
| 100 µM vs. Ctrl | 33.94, 1 | 5.826 | <0.0001 | **** | Two-sided | Yes |
| 1 mM vs. Ctrl | 5.889, 1 | 2.427 | 0.0152 | * | Two-sided | Yes |
| 10 mM vs. Ctrl | 13.79, 1 | 3.713 | 0.0002 | *** | Two-sided | Yes |

**Table 7:** Contingency statistical results of cutoff R-R values of aminophylline treated HL-1 versus control.

| Chi-square with Yates' correction | Chi-square, df | z | P value | P value summary | One- or two-sided | Statistically significant (P < 0.05)? |
| --- | --- | --- | --- | --- | --- | --- |
| 8 µM vs. Ctrl | 10.44, 1 | 3.231 | 0.0012 | ** | Two-sided | Yes |
| 16 µM vs. Ctrl | 177.2, 1 | 13.31 | <0.0001 | **** | Two-sided | Yes |
| 32 µM vs. Ctrl | 129.6, 1 | 11.38 | <0.0001 | **** | Two-sided | Yes |
| 64 µM vs. Ctrl | 46.77, 1 | 6.839 | <0.0001 | **** | Two-sided | Yes |
| 128 µM vs. Ctrl | 159.3, 1 | 12.62 | <0.0001 | **** | Two-sided | Yes |
| 256 µM vs. Ctrl | 3.207, 1 | 1.791 | 0.0733 | ns | Two-sided | No |
| 512 µM vs. Ctrl | 244.0, 1 | 15.62 | <0.0001 | **** | Two-sided | Yes |

**Figure 1:** Example of effects of heart modulators (isoproterenol, non-selective β adrenoceptor agonist, and metoprolol β adrenoceptor blocker) on hPSC-CMs and HL-1 (only isoproterenol) cellular lines. *Scatter plots (mean ± standard deviation) of BR overall changes, normalized to a baseline measurement (relative response) and to control measurements means (n = 4 for Metoprolol 70 µM and isoproterenol 1 µM, n=3 isoproterenol 0.1 µM, n=9 for hPSC-CMs ctrl’s and n=5 for HL-1 ctrl). At least three biological repetitions were used in each column. Brown-Forsythe and Welch ANOVA test was used for statistical analysis (ctrl vs. metoprolol 70 µM p=0.016, ctrl vs. isoproterenol p=0.0015 and ctrl vs.isoproterenol p=0.039).*

**
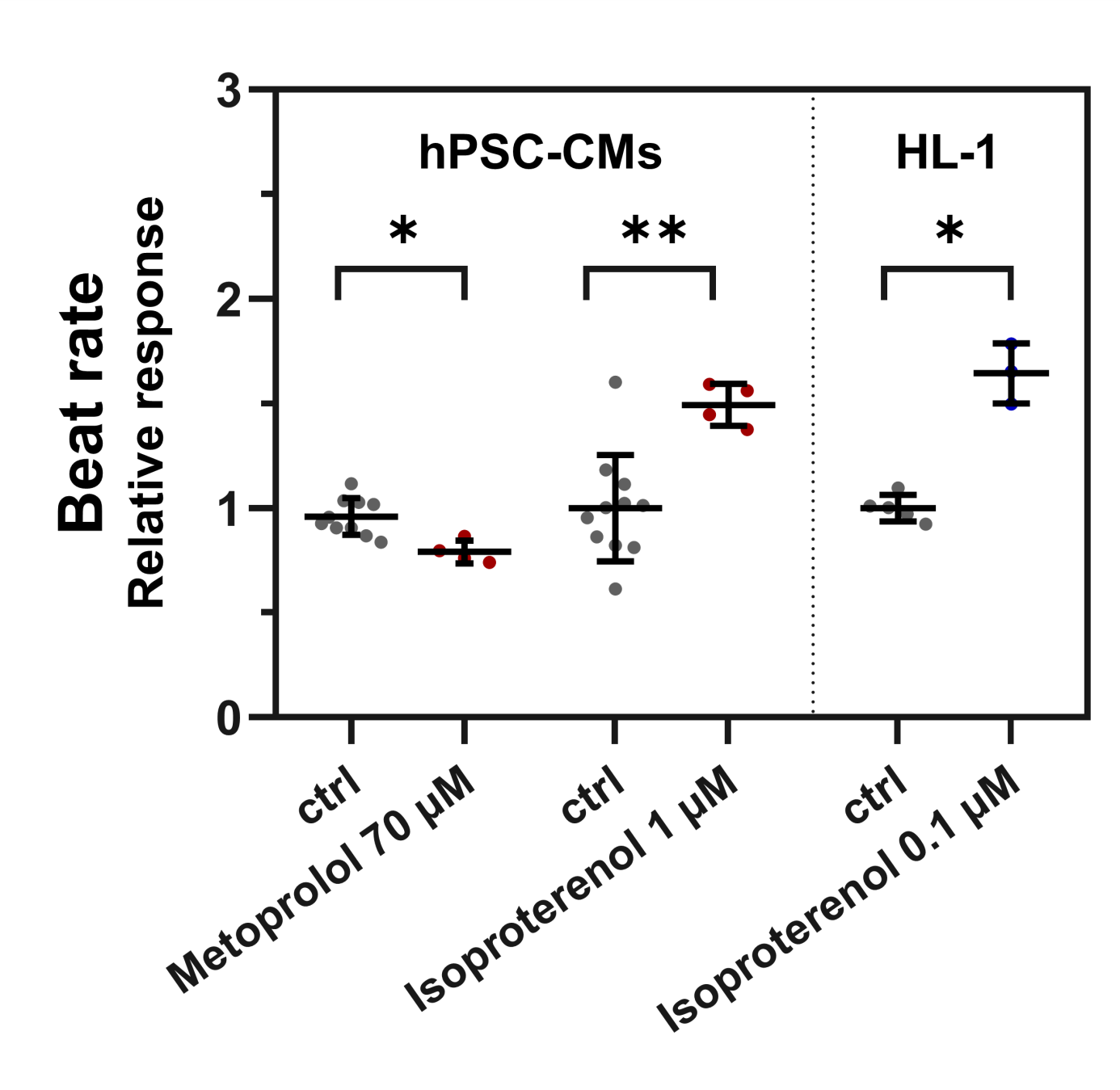
**

**Figure 2:** Linear regression of BR (A; blue dots) and contraction force (C; red dots) relative responses and measured concentrations coupled with respective residual plots (B, D). In case of beat rate, results show statistically significant non-zero slope (Walt test, p<0.0001) proving positive relationship between concentration and relative response. Similar relationship was not found in case of contraction force.


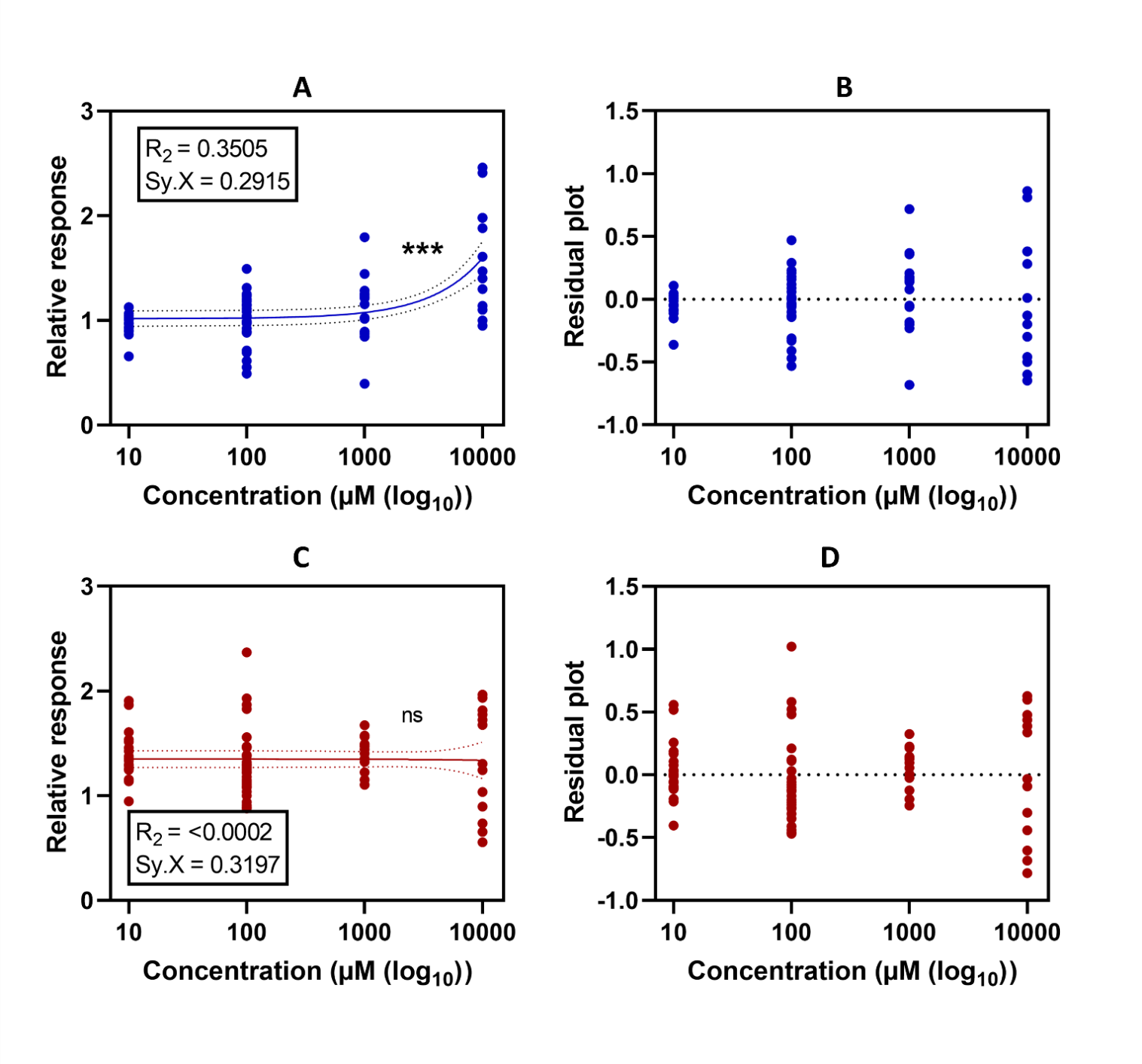


*
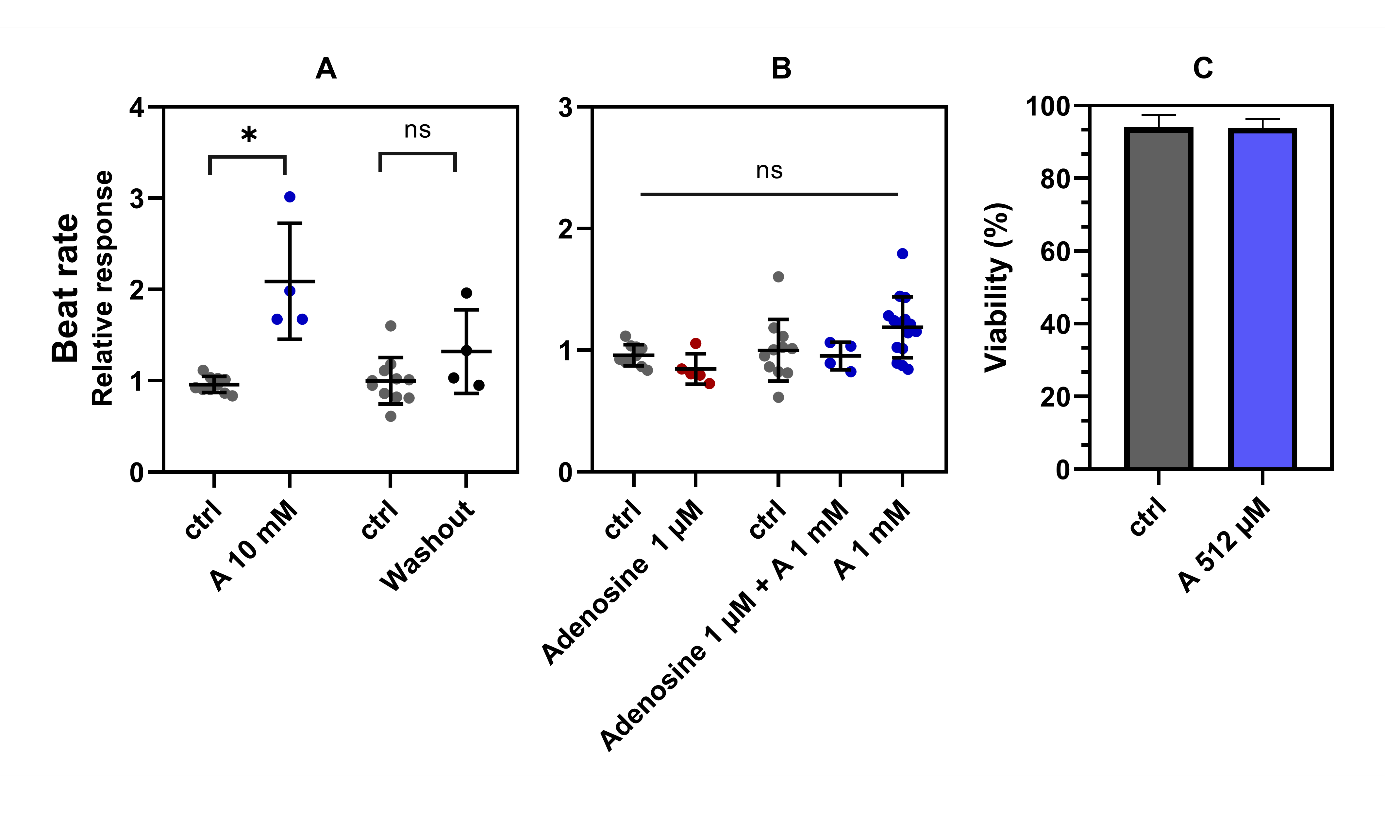
***Figure 3:** Control experiments. *Scatter plots (mean ± standard deviation) of BR overall changes, normalized to a baseline measurement (relative response) and to control measurements means.* ***(A)*** *hPSC-CMs treated with 10 mM aminophylline (A 10mM) followed by washout period with Tyrode medium. It is visible that elevated BR as a effect of aminophylline ceased during a washout period (n=9 for ctlr’s, n=4 for A 10 mM and washout; Brown-Forsythe and Welch ANOVA test).* ***(B)*** *hPSC-CMs treated with 1 µM adenosine followed by combination of 1 µM adenosine and 1 mM aminophylline (A 1 mM). Results showed no significant differences compared to controls (n=9 for ctlr’s, n=5 for 1 µM adenosine and n=4 1 µM adenosine + A 1 mM; Brown-Forsythe and Welch ANOVA test).* ***(C)*** *Results of viability assay of HL-1 cells showing no significant differences between cells treated with 512 µM aminophylline (A 512 µM) and control (n=3 for ctlr, n=5 for A 512 µM).*
